# Antimicrobial resistance in India's dairy value chain: An investigation of risk factors in Haryana and Assam, India

**DOI:** 10.1016/j.onehlt.2026.101391

**Published:** 2026-03-20

**Authors:** Tushar K. Dey, Garima Sharma, Åke Lundkvist, Delia Grace, Bibek R. Shome, Ram P. Deka, Rajeswari Shome, Samiran Bandyopadhyay, Naresh K. Goyal, Johanna F. Lindahl

**Affiliations:** a Department of Biosciences, International Livestock Research Institute, Nairobi 00100, Kenya; b Zoonosis Science Center, Department of Medical Biochemistry and Microbiology, Uppsala University, 75123 Uppsala, Sweden; c ICAR-National Institute of Veterinary Epidemiology and Disease Informatics, Bengaluru 560064, India; d Department of Clinical Sciences, Swedish University of Agricultural Sciences, 75007 Uppsala, Sweden; e Food and Markets Department, Natural Resources Institute, Chatham Maritime ME4 4TB, UK; f International Livestock Research Institute, Regional Office for South Asia, New Delhi 110012, India; g Eastern Regional Station, ICAR-Indian Veterinary Research Institute, Kolkata 700037, India; h Dairy Microbiology Division, National Dairy Research Institute, Karnal 132001, India

**Keywords:** Antimicrobial resistance, Dairy, Multidrug resistance, One health, Pasteurized milk, Raw milk

## Abstract

Antimicrobial resistance (AMR) in food products represents a potential public health risk in low- and middle-income countries, yet evidence from Indian milk production systems remains limited. A cross-sectional study was conducted using 256 milk samples collected from farms and vendors in Assam and Haryana, India. AMR prevalence was high (90%) with no state-level differences. Dual AMR (both Gram-positive and Gram-negative bacteria) occurred in 48% [95% confidence interval (CI) 42–55] of samples, while multidrug resistance (MDR) was also 48% [CI 42–55], but were not significant. Dual AMR was only present in 7% of samples [CI 4–11]. AMR bacteria were detected in milk from both informal and more organised dairy systems. In multivariable analyses, none of the investigated farm-level or vendor-level predictors showed strong or consistent associations with AMR outcomes after adjustment. However, due to limited statistical power and sparse outcomes, the observed associations should be interpreted cautiously. The findings are primarily descriptive and highlight the need for larger, systematically designed studies to better understand drivers of AMR along the Indian dairy value chain.

## Highlights

• AMR is widespread in milk from Assam and Haryana, India, including multidrug resistant isolates.
• Resistance in both Gram-negative and Gram-positive bacteria detected pasteurized milk samples.
• Risk-factor analyses could not identify any farm level risk factors.
• Results highlight the complexity in understanding drivers of AMR along a value chain.

## 1 Introduction

Antimicrobial resistance (AMR) has emerged as a major global health concern, particularly in agricultural and dairy sectors, where extensive antibiotic use contributes to the emergence and spread of resistant pathogens [1], [2], [3], [4]. The livestock and dairy sectors are increasingly recognized as important components of the AMR landscape, given their reliance on antibiotics for disease management and productivity.

India is the world's largest producer of milk, with nearly 80% of production originating from smallholder dairy farmers [5]. Milk is an important source of animal-derived protein, particularly in countries such as India where a large proportion of the population follows a vegetarian diet, and the dairy sector also plays a crucial role in rural livelihoods by generating income and employment for millions of households involved in the milk value chain [6]. A substantial share of milk in India is marketed through the unorganized dairy sector [7], [8], where traditional milkmen or vendors collect milk from individual farmers and sell it directly to consumers [9], [10]. Milk distributed through these informal channels is frequently raw and unprocessed, whereas the formal dairy sector, consisting of cooperatives and private companies, supplies pasteurized and packaged milk [11]. While the informal sector offers important economic and social benefits, it also raises significant food safety concerns [12]. Smallholder dairy farms often operate with limited resources, inadequate infrastructure, and restricted access to veterinary and animal health services [13], [14], [15]. Under such conditions, milk can serve as a vehicle for the transmission of foodborne pathogens from animals to humans [16].

Antibiotics play an important role in dairy production by helping control infectious diseases and maintain animal health, thereby sustaining productivity. In India and other low- and middle-income countries (LMICs), antibiotics are frequently used not only for therapeutic purposes but also prophylactically or metaphylactically to prevent infections and improve growth and production [17], [18], [19]. Although antibiotics can enhance animal health and productivity, their misuse or overuse may facilitate the emergence and spread of AMR bacteria [20]. The irrational and non-therapeutic use of antibiotics in farm animals can therefore have substantial implications for public health.

AMR in bacteria isolated from dairy animals and milk represents a significant One Health challenge due to the zoonotic potential of milk-borne pathogens and their resistance determinants [21].

Both raw and pasteurized milk may harbour AMR bacteria and resistance genes, posing risks to consumer safety and complicating treatment outcomes [22]. Raw milk, in particular, is recognized as a reservoir of AMR in both Gram-positive (GPB) and Gram-negative bacteria (GNB). Pathogens such as *Staphylococcus aureus, Escherichia coli, Klebsiella pneumoniae,* and *Salmonella* spp. have frequently been isolated from raw milk, with several studies reporting multidrug resistance (MDR) among these organisms [20], [23], [24], [25], [26]. Consequently, AMR in dairy production systems not only affects animal health but also poses zoonotic risks to humans through contaminated milk and dairy products [27], [28], [29].

Beyond direct antibiotic usage, indirect pathways may also contribute to the emergence and spread of AMR. Antibiotics administered for treatment of disease, such as mastitis therapy, can persist in the environment and contaminate water, soil, and feed sources, thereby promoting the selection of resistant bacteria outside the animal host [17], [30]. The dairy production environment thus represents a complex system where environmental, management, and biological factors interact to influence the development and dissemination of AMR [31]. Previous studies have identified factors such as herd size, farm hygiene practices, and disease prevalence as potential contributors to AMR occurrence [31], [32]. However, the role of farmer knowledge and antibiotic stewardship practices remains insufficiently explored in the Indian dairy context.

To understand the AMR variation in dairy production system, the study was conducted in two geographically distant Indian states, Assam and Haryana, separated by approximately 2300 km.

Assam is located in north-eastern India (26.2006° N, 92.9376° E), along the Brahmaputra River, and shares international borders with Bhutan and Bangladesh. The state has a humid subtropical climate with hot and humid summers, mild winters, and a pronounced monsoon season from June to September. According to the Economic Survey of the Government of Assam from the 20th Livestock Census (2019), the state has approximately 10.9 million cattle and 0.4 million buffaloes, with an estimated annual milk production of 975 million litres [33].

Haryana, located in northern India (29.0588° N, 76.0586° E), has a semi-arid climate characterised by hot summers, cool winters and relatively low annual rainfall. According to the 2019 Livestock Census, the state has approximately 1.93 million cattle and 4.38 million buffaloes and produces an estimated 10.63 million tonnes of milk annually.

Common bovine diseases reported in both states include foot-and-mouth disease, mastitis and brucellosis. Although both states are important milk-producing regions, their dairy production systems differ substantially. Haryana represents a highly developed and organised dairy sector dominated by commercial and cooperative farms, whereas Assam is characterised by a less developed and predominantly unorganized dairy system.

To address this gap, the present study aims to identify key predictors of AMR in dairy farms. Based on existing evidence and the proposed causal structure, we hypothesized that: (i) milk samples originating from different dairy production settings (Assam vs. Haryana) differ in their probability of containing AMR bacteria; (ii) factors such as reported antibiotic use, mastitis history, herd size, cow hygiene, and farmers' knowledge are associated with the presence of AMR and MDR in milk samples; and (iii) samples containing resistant GPB are more likely to also harbour resistant GNB (dual AMR). These hypotheses informed the construction of the directed acyclic graph and the selection of covariates for regression analyses.

## 2 Methods

### 2.1 Ethics statement

The study received ethical clearance from the Institutional Research Ethics Committee (IREC) of the International Livestock Research Institute (ILRI) on 21 September 2015 (No. ILRI-IREC2015–12) and 27 February 2017 (No. ILRI-IREC2017–05), with additional approval from collaborating institutes under the Indian Council of Agricultural Research (ICAR). This study did not involve the use of live animals; all milk samples were sourced from farmers and vendors. Consequently, the ARRIVE guidelines [34] are not applicable to this research.

### 2.2 Study design

**Fig. 1:**
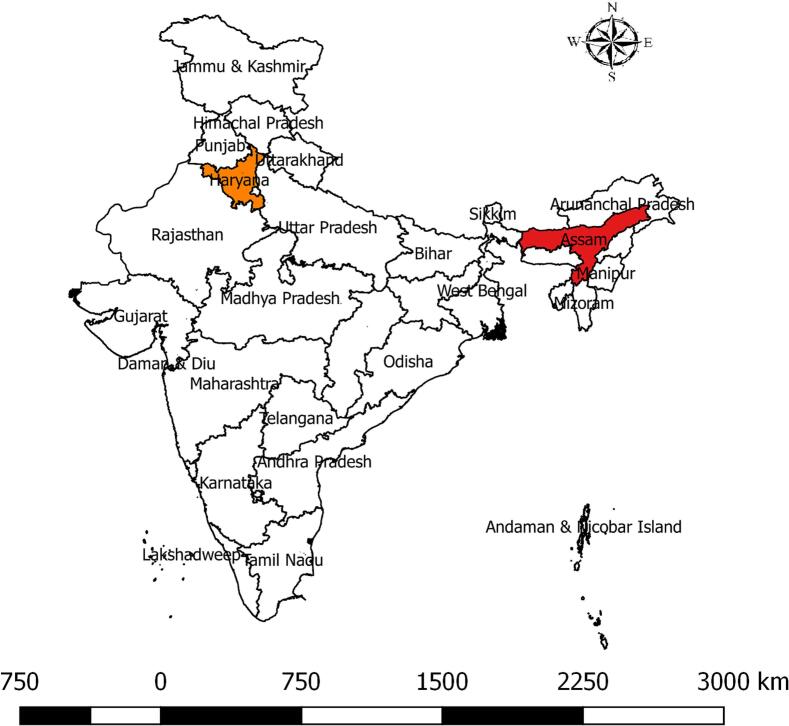
Geographic distribution of sampling sites in India, highlighting Assam (red) and Haryana (orange). (For interpretation of the references to colour in this figure legend, the reader is referred to the web version of this article.)

A cross-sectional study was conducted in Assam and Haryana (Fig. 1), selected to represent contrasting dairy production systems. Assam represents a predominantly informal dairy sector dominated by local cattle breeds, whereas Haryana represents a more organised dairy system with high-yielding cattle and buffalo.

The sampling and microbiological procedures followed standard laboratory protocols and were consistent with those used in our previous related studies [24], [35]. Milk samples were processed to isolate GPB and GNB. For GPB, samples were enriched in mannitol salt broth and cultured on staphylococci agar S110 (Hi-media, Maharashtra, India) for presumptive staphylococci isolation, followed by characterization using colony morphology, Gram staining, mannitol fermentation, pigment production, and gelatinase activity. Methicillin resistance was screened using disk diffusion, with resistant isolates tested for *mecA* and *mecC* genes by PCR, followed by species identification and MIC determination using *E*-tests.

For GNB, samples were enriched in buffered peptone water and plated on MacConkey and Eosin Methylene Blue (EMB) agar (Hi-media) to isolate presumptive *E. coli, Shigella*, and *Klebsiella*. Colonies were characterised by morphology, Gram staining, and lactose fermentation. Antibiotic susceptibility was assessed by disk diffusion to detect ESBL, AmpC, and MBL phenotypes, followed by PCR screening for β-lactamase genes. Confirmed isolates were identified to species level and MICs were determined using *E*-tests. Further PCR genotyping was conducted to identify specific AMR genes and bacterial species. *Staphylococcus aureus* ATCC 25923 was used as the quality control for GPB, while *Escherichia coli* ATCC 25922 served as the quality control for GNB.

This methodological approach resulted in the recovery of 256 GPB isolates from the milk samples. At the genus level, most GPB isolates were identified as *Staphylococcus* spp. Only 16 isolates were further characterised to the species level using PCR, including *Staphylococcus arlettae* (*n* = 1), *Staphylococcus aureus* (*n* = 5), *Staphylococcus epidermidis* (n = 5), *Staphylococcus pseudoxylosus* (*n* = 2), *Staphylococcus sciuri* (n = 1), and *Staphylococcus warneri* (n = 1). Among these, 15 isolates belonged to the genus *Staphylococcus*, while one isolate was identified as *Enterococcus gallinarum* based on partial 16S rDNA sequencing. The remaining 240 *Staphylococcus* isolates were not identified to the species level because they did not carry the methicillin-resistance genes (*mecA/mecC*), and further characterization of mec-negative isolates was beyond the scope of this study.

Similarly, this methodological approach resulted in the recovery of 256 GNB isolates from the milk samples. Among these, 35 isolates were confirmed as β-lactamase gene carriers by PCR. Of the β-lactamase-positive isolates, 14 were identified to the genus level as *Klebsiella* spp. (*n* = 5), *Shigella* spp. (*n* = 7), and *Escherichia coli* (*n* = 2). The remaining 21 β-lactamase-positive isolates could not be assigned to these genera and were therefore classified as other resistant GNB. Isolates that were negative for β-lactamase genes were not further identified and were retained as GNB for the purpose of sample-level analyses.

In the present analysis, only milk samples for which AMR results were available [including methicillin-resistance and beta-lactam resistance (ESBL, MBL, AmpC)] for both GPB and GNB were included. In total, 256 milk samples were thus included, comprising 156 samples from Haryana and 100 samples from Assam.

**Table 1:** Samples from milk, categorized by state and source.

State	Milk samples	Milk from farmers	Milk from vendors	Raw milk	Pasteurized milk
Assam	100	37	63	100	0
Haryana	156	66	90	139	17
Total	256	103	153	239	17

Overall, 103 samples were obtained directly from dairy farmers and 153 from milk vendors. Among vendor samples, 17 were pasteurized milk purchased from retail outlets (Haryana only), while the remaining vendor samples were raw milk (supplementary material 1) (Table 1).

In both states, dairy farmers and vendors were informed about the study and interviewed using a structured questionnaire to assess their knowledge, attitudes and practices related to antimicrobial use, milk safety and hygiene (supplementary material 2) [22], [36].

### 2.3 Definition of resistance outcomes

Methicillin resistance in GPB was assessed in two steps. Isolates were classified as phenotypically methicillin-resistant if they showed resistance to one or both β-lactam antibiotics (oxacillin and cefoxitin) using the disc diffusion test (DDT). Genotypic methicillin resistance was confirmed by PCR detection of the *mecA* or *mecC* genes.

For GNB, phenotypic resistance patterns were used to classify isolates as extended-spectrum β-lactamase (ESBL) producers (resistant to cefotaxime and ceftazidime), AmpC β-lactamase producers (resistant to cefoxitin and cefotetan) and metallo-β-lactamase (MBL) producers (resistant to imipenem and meropenem). Isolates exhibiting resistance to any single antibiotic tested by DDT were classified as resistant, irrespective of antibiotic class.

PCR detection of β-lactamase genes was used to confirm genotypic β-lactamase production, and isolates were further identified as *Shigella* spp., *Klebsiella* spp. or *Escherichia coli*, while the remaining isolates were grouped as other Enterobacteriaceae.

For the purpose of this analysis, GPB isolates were classified as MDR if they were resistant to both oxacillin and cefoxitin, whereas GNB isolates were classified as MDR if they were resistant to three or more antibiotics.

For risk-factor analyses, a milk sample was classified as resistant if either the GPB or the GNB component was resistant to at least one antibiotic. Dual AMR was defined when both GPB and GNB isolated from the same sample exhibited phenotypic resistance. A sample was classified as MDR if either GPB (resistant to both oxacillin and cefoxitin) or GNB (resistant to three or more antibiotics) fulfilled the MDR definition, and as dual MDR if both GPB and GNB were MDR.

All isolates were categorized as resistant, intermediate or susceptible based on the Kirby–Bauer disc diffusion method in accordance with the guidelines of the Clinical and Laboratory Standards Institute [37], [38], [39], [40].

### 2.4 Data management and statistical analysis

Data were initially entered into Microsoft Excel (Version 2308, Build 16.0.16731.20052) and subsequently analysed using Stata version 15.1 (StataCorp, College Station, TX, USA). Descriptive statistics were generated for all variables. Categorical variables were summarised as frequencies and percentages together with 95% confidence intervals (CIs). Associations between categorical variables were assessed using the chi-square test or Fisher's exact test, as appropriate. All statistical tests were two-sided, and a *p*-value <0.05 was considered statistically significant.

**Fig. 2:**
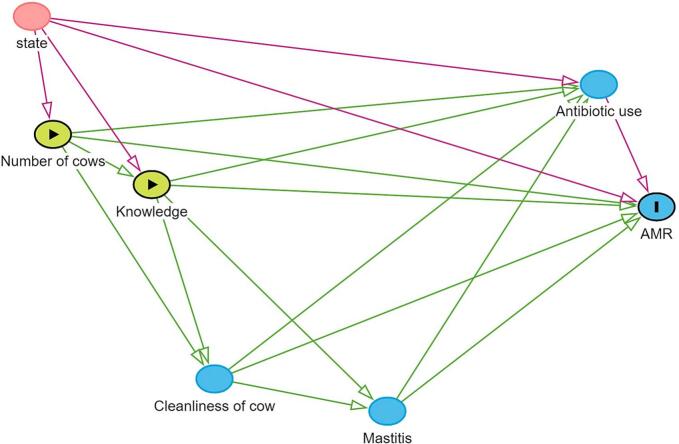
Causal diagram depicting the outcome of interest (presence of antimicrobial resistance; AMR) and proposed risk factors in milk samples from farms. Green arrows represent hypothesized causal effects, whereas pink arrows represent pathways that are mediating, indirect or context dependent. (For interpretation of the references to colour in this figure legend, the reader is referred to the web version of this article.)

A directed acyclic graph (DAG; Fig. 2), developed using DAGitty, was used to guide the analytical framework and the selection of covariates for regression analyses.

The primary outcomes were defined at the sample level as:

(i) presence of antimicrobial-resistant (AMR) bacteria in a sample (either GPB or GNB), (ii) dual AMR, defined as the presence of AMR in both GPB and GNB within the same sample, and (iii) presence of multidrug-resistant (MDR) isolates in a sample.

Logistic regression models were used to evaluate associations between each outcome and potential predictors, including state, herd size, history of mastitis, reported antibiotic use, cow hygiene and farmers' knowledge level. Both univariable and multivariable logistic regression models were fitted, and odds ratios (ORs) with 95% CIs were reported. Missing data were handled using complete-case (listwise) deletion.

Variables were selected for inclusion in multivariable models a priori based on biological plausibility and relevance to the study objectives, as informed by the DAG. In addition, variables showing evidence of association in univariable analyses were considered for inclusion. Final multivariable models were constructed based on conceptual relevance and data availability rather than solely on statistical significance in univariable screening.

Farmers' knowledge was measured using 11 questions related to antibiotic use and antimicrobial resistance. Responses were dichotomised as correct (1) or incorrect (0), summed to obtain a total knowledge score, following approach used in our previous study [28] and included as a predictor.

Model selection and comparison were supported by assessment of model fit using the Akaike Information Criterion (AIC). To account for small-sample bias and potential quasi-complete separation in logistic regression models, Firth's penalized maximum likelihood estimation was applied using the firthlogit command in Stata. Model diagnostics included assessment of multicollinearity, goodness-of-fit and underlying model assumptions.

The diagram highlights multiple routes through which AMR may develop, identifying antibiotic use as a central driver. Farmers' knowledge and cow cleanliness represent potential intervention points that may indirectly mitigate AMR. Herd size (number of cows) appears as an upstream driver influencing several other variables and thereby affecting AMR both directly and indirectly.

## 3 Results

### 3.1 Identification of bacteria

Prevalence of resistance in either GPB or GNB was uniformly high across both states, with 90% of samples having a resistant bacterium present (Table 2).

**Table 2:** Antimicrobial resistance by state.

AMR presence in samples				
State	Present n (%) [95% CI]	Not Present n (%) [95% CI]	Total n	*p*-value
Assam	91 (91%) [84–96]	9 (9%) [4], [5], [6], [7], [8], [9], [10], [11], [12], [13], [14], [15], [16]	100	0.395^1^
Haryana	139 (89%) [83–93]	17 (11%) [6], [7], [8], [9], [10], [11], [12], [13], [14], [15], [16], [17]	156	
Total	230 (90%) [85–93]	26 (10%) [7], [8], [9], [10], [11], [12], [13], [14]	256	
Dual AMR presence in both Gram± bacteria in samples				
State	Present n (%) [95% CI]	Not Present n (%) [95% CI]	Total n	*p*-value
Assam	54 (54%) [44–64]	46 (46%) [36], [37], [38], [39], [40], [41], [42], [43], [44], [45], [46], [47], [48], [49], [50], [51], [52], [53], [54], [55], [56]	100	0.097^1^
Haryana	70 (45%) [37], [38], [39], [40], [41], [42], [43], [44], [45], [46], [47], [48], [49], [50], [51], [52], [53]	86 (55%) [47–63]	156	
Total	124 (48%) [42], [43], [44], [45], [46], [47], [48], [49], [50], [51], [52], [53], [54], [55]	132 (52%) [45], [46], [47], [48], [49], [50], [51], [52], [53], [54], [55], [56], [57], [58]	256	
MDR presence in samples				
State	Present n (%) [95% CI]	Not Present n (%) [95% CI]	Total n	*p*-value
Assam	45 (45%) [35], [36], [37], [38], [39], [40], [41], [42], [43], [44], [45], [46], [47], [48], [49], [50], [51], [52], [53], [54], [55]	55 (55%) [45–65]	100	0.226^1^
Haryana	79 (51%) [42], [43], [44], [45], [46], [47], [48], [49], [50], [51], [52], [53], [54], [55], [56], [57], [58], [59]	77 (49%) [41], [42], [43], [44], [45], [46], [47], [48], [49], [50], [51], [52], [53], [54], [55], [56], [57]	156	
Total	124 (48%) [42], [43], [44], [45], [46], [47], [48], [49], [50], [51], [52], [53], [54], [55]	132 (52%) [45], [46], [47], [48], [49], [50], [51], [52], [53], [54], [55], [56], [57], [58]	256	
Dual MDR presence in samples (both Gram± bacteria)				
State	Present n (%) [95% CI]	Not Present n (%) [95% CI]	Total n	*p*-value
Assam	5 (5%) [2], [3], [4], [5], [6], [7], [8], [9], [10], [11]	95 (95%) [89–98]	100	0.224^1^
Haryana	13 (8%) [4], [5], [6], [7], [8], [9], [10], [11], [12], [13], [14]	143 (92%) [87–95]	156	
Total	18 (7%) [4], [5], [6], [7], [8], [9], [10], [11]	238 (93%) [89–96]	256	

“Present” indicates that at least one resistant bacterial isolate was detected in the sample. “Not present” indicates that no resistant bacterial isolate was detected in the sample (i.e., bacteria may have been isolated, but none showed resistance to the tested antimicrobials). 95% confidence intervals were calculated using binomial exact methods. ^1^One-sided Fisher's exact test.

For dual AMR (resistance in both GPB and GNB), prevalence was slightly higher in Assam [54%, 54/100, CI (44–64)] than in Haryana [45%, 70/156, CI (37–53), but the difference did not reach statistical significance (*p* = 0.097). The presence of MDR in samples was 45% [45/100, CI (35–55)] in Assam and 51% [79/156, CI (42–59)] in Haryana, and dual MDR prevalence remained low in both states, with 5% [5/100, CI (2−11)] in Assam and 8% [13/156, CI (4–14)] in Haryana.

Overall, AMR was high across milk types and sources, but dual MDR was significantly common in pasteurized milk compared to raw milk, while vendor-farmer differences were not significant (Table 3). Pasteurized milk had 94% AMR [16/17, CI (71–100)] compared to 90% in raw milk [214/239, CI (85–93)], but the difference was not statistically significant (*p* = 0.466). Similarly, farmer-sourced milk had 92% AMR [95/103, CI (85–96)] versus 88% in vendor samples [135/153, CI (82–93)], with no significant difference (*p* = 0.205) (Table 3).

**Table 3:** Sample-level prevalence of antimicrobial resistance (AMR), dual AMR, multidrug resistance (MDR), and dual MDR by milk type and sample source.

AMR presence in samples (either Gram± bacteria) by milk type				
Milk Type	Present n (%) [95% CI]	Not Present n (%) [95% CI]	Total n	*p*-value
Pasteurized milk	16 (94%) [71–100]	1 (6%) [0.1–29]	17	0.466^1^
Raw milk	214 (90%) [85–93]	25 (10%) [7], [8], [9], [10], [11], [12], [13], [14], [15]	239	
Total	230 (90%) [85–93]	26 (10%) [7], [8], [9], [10], [11], [12], [13], [14]	256	
AMR presence in samples (either Gram± bacteria) by sample source				
Sample Source	Present n (%) [95% CI]	Not Present n (%) [95% CI]	Total n	*p*-value
Farmer	95 (92%) [85–96]	8 (8%) [3], [4], [5], [6], [7], [8], [9], [10], [11], [12], [13], [14], [15]	103	0.205^1^
Vendor	135 (88%) [82–93]	18 (12%) [7], [8], [9], [10], [11], [12], [13], [14], [15], [16], [17], [18]	153	
Total	230 (90%) [85–93]	26 (10%) [7], [8], [9], [10], [11], [12], [13], [14]	256	
Dual AMR presence in samples (both Gram± bacteria) by milk type				
Milk Type	Present n (%) [95% CI]	Not Present n (%) [95% CI]	Total n	*p*-value
Pasteurized milk	9 (53%) [28–77]	8 (47%) [23–72]	17	0.446^1^
Raw milk	115 (48%) [42], [43], [44], [45], [46], [47], [48], [49], [50], [51], [52], [53], [54], [55]	124 (52%) [45], [46], [47], [48], [49], [50], [51], [52], [53], [54], [55], [56], [57], [58]	239	
Total	124 (48%) [42], [43], [44], [45], [46], [47], [48], [49], [50], [51], [52], [53], [54], [55]	132 (52%) [45], [46], [47], [48], [49], [50], [51], [52], [53], [54], [55], [56], [57], [58]	256	
Dual AMR presence in samples (both Gram± bacteria) by sample source				
Sample Source	Present n (%) [95% CI]	Not Present n (%) [95% CI]	Total n	*p*-value
Farmer	53 (51%) [41], [42], [43], [44], [45], [46], [47], [48], [49], [50], [51], [52], [53], [54], [55], [56], [57], [58], [59], [60], [61]	50 (49%) [38], [39], [40], [41], [42], [43], [44], [45], [46], [47], [48], [49], [50], [51], [52], [53], [54], [55], [56], [57], [58], [59]	103	0.253^1^
Vendor	71 (46%) [38], [39], [40], [41], [42], [43], [44], [45], [46], [47], [48], [49], [50], [51], [52], [53], [54], [55]	82 (54%) [45], [46], [47], [48], [49], [50], [51], [52], [53], [54], [55], [56], [57], [58], [59], [60], [61], [62]	153	
Total	124 (48%) [42], [43], [44], [45], [46], [47], [48], [49], [50], [51], [52], [53], [54], [55]	132 (52%) [45], [46], [47], [48], [49], [50], [51], [52], [53], [54], [55], [56], [57], [58]	256	
MDR presence in samples (either Gram± bacteria) by milk type				
Milk Type	Present n (%) [95% CI]	Not Present n (%) [95% CI]	Total n	*p*-value
Pasteurized milk	9 (53%) [28–77]	8 (47%) [23–72]	17	0.446^1^
Raw milk	115 (48%) [42], [43], [44], [45], [46], [47], [48], [49], [50], [51], [52], [53], [54], [55]	124 (52%) [45], [46], [47], [48], [49], [50], [51], [52], [53], [54], [55], [56], [57], [58]	239	
Total	124 (48%) [42], [43], [44], [45], [46], [47], [48], [49], [50], [51], [52], [53], [54], [55]	132 (52%) [45], [46], [47], [48], [49], [50], [51], [52], [53], [54], [55], [56], [57], [58]	256	
MDR presence in samples (either Gram± bacteria) by sample source				
Sample Source	Present n (%) [95% CI]	Not Present n (%) [95% CI]	Total n	*p*-value
Farmer	55 (53%) [43–63]	48 (47%) [37], [38], [39], [40], [41], [42], [43], [44], [45], [46], [47], [48], [49], [50], [51], [52], [53], [54], [55], [56], [57]	103	0.120^1^
Vendor	69 (45%) [37], [38], [39], [40], [41], [42], [43], [44], [45], [46], [47], [48], [49], [50], [51], [52], [53]	84 (55%) [47–63]	153	
Total	124 (48%) [42], [43], [44], [45], [46], [47], [48], [49], [50], [51], [52], [53], [54], [55]	132 (52%) [45], [46], [47], [48], [49], [50], [51], [52], [53], [54], [55], [56], [57], [58]	256	
Dual MDR presence in samples (both Gram± bacteria) by milk type				
Milk Type	Present n (%) [95% CI]	Not Present n (%) [95% CI]	Total n	*p*-value
Pasteurized milk	4 (23%) [7], [8], [9], [10], [11], [12], [13], [14], [15], [16], [17], [18], [19], [20], [21], [22], [23], [24], [25], [26], [27], [28], [29], [30], [31], [32], [33], [34], [35], [36], [37], [38], [39], [40], [41], [42], [43], [44], [45], [46], [47], [48], [49], [50]	13 (76%) [50–93]	17	0.023^1^
Raw milk	14 (6%) [3], [4], [5], [6], [7], [8], [9], [10]	225 (94%) [90–97]	239	
Total	18 (7%) [4], [5], [6], [7], [8], [9], [10], [11]	238 (93%) [89–96]	256	
Dual MDR presence in samples (both Gram± bacteria) by sample source				
Sample Source	Present n (%) [95% CI]	Not Present n (%) [95% CI]	Total n	*p*-value
Farmer	8 (8%) [3], [4], [5], [6], [7], [8], [9], [10], [11], [12], [13], [14], [15]	95 (92%) [85–96]	103	0.444^1^
Vendor	10 (7%) [3], [4], [5], [6], [7], [8], [9], [10], [11], [12]	143 (93%) [88–97]	153	
Total	18 (7%) [4], [5], [6], [7], [8], [9], [10], [11]	238 (93%) [89–96]	256	

“Present” indicates that at least one resistant bacterial isolate was detected in the sample. “Not present” indicates that no resistant bacterial isolate was detected in the sample (i.e., bacteria may have been isolated, but none showed resistance to the tested antimicrobials). ***Dual AMR and dual MDR indicate the presence of resistant or multidrug-resistant bacteria in both Gram-positive and Gram-negative isolates recovered from the same sample.*** 95% confidence intervals were calculated using binomial exact methods. ^1^One-sided Fisher's exact test.

For dual AMR (sample harbouring both resistant GPB and GNB), the prevalence was 53% in pasteurized milk [9/17, CI (28–77)] and 48% in raw milk [115/239, CI (42–55)], with no significant difference (*p* = 0.446). Farmer samples showed 51% dual AMR [53/103, CI (41–61)] compared to 46% in vendor samples [71/153, CI (38–55), but non-significant (*p* = 0.253).

Herd size (number of milking cows) was not significantly associated with AMR or MDR. Odds ratios were close to 1 with wide confidence intervals, indicating no clear relationship. Cleanliness of cows appeared to increase the odds of dual AMR (OR = 3.74, 95% CI: 1.13–12.39, *p* = 0.031), suggesting a potential association. However, results for other AMR/MDR outcomes were inconsistent, and confidence intervals were wide, reflecting uncertainty.

History of mastitis and antibiotic use were not significant predictors of AMR or MDR. While odds ratios for mastitis and antibiotic use showed directions toward higher MDR, the estimates were imprecise and confidence intervals crossed unity (Table 4).

**Table 4:** Univariable analysis of antimicrobial resistance (AMR) and multidrug resistance (MDR) predictors.

Predictors	AMR						MDR					
	AMR samples (either Gram± bacteria)			AMR samples (both Gram± bacteria)			MDR samples (either Gram± bacteria)			MDR samples (both Gram± bacteria)		
State	Odds ratio	95% CI	*p*-value	Odds ratio	95% CI	*p*-value	Odds ratio	95% CI	*p*-value	Odds ratio	95% CI	*p*-value
Haryana	0.81	0.35–1.89	0.624	0.69	0.42–1.15	0.155	1.25	0.76–2.08	0.379	1.73	0.60–5.00	0.314
Assam	Reference			Reference			Reference			Reference		
Constant	10.11	5.10–20.06	<0.001	1.17	0.79–1.74	0.424	0.82	0.55–1.21	0.318	0.05	0.021–0.13	<0.001
Milking cow	1.28	0.54–3.03	0.567	1.10	0.92–1.32	0.270	1.08	0.92–1.27	0.367	0.10⁎	0.76-1.27	0.916
Constant	7.92	1.97–31.87	0.004	0.86	0.52–1.42	0.550	1.01	0.62–1.65	0.961	0.10⁎	0.04-0.23	<0.001
Cleanliness of cow	3.79⁎	0.21–68.80	0.368	3.74	1.13–12.39	0.031	1.30	0.45–3.74	0.624	0.71	0.08–6.13	0.752
Constant	9.24⁎	4.55-18.74	<0.001	0.87	0.57–1.33	0.518	1.10	0.72–1.68	0.666	0.09	0.04–0.19	<0.001
History of Mastitis	1.31	0.15–11.46	0.806	1.71	0.57–5.10	0.340	2.15	0.69–6.71	0.187	1.93	0.35–10.53	0.448
Constant	11.43	5.28–24.75	<0.001	0.98	0.64–1.49	0.915	1.02	0.67–1.56	0.915	0.07	0.032–0.17	<0.001
Antibiotic use	0.54	0.06–5.05	0.589	1.31	0.28–6.15	0.735	2.35	0.43–12.70	0.321	0.70⁎	0.04-13.38	0.814
Constant	11.12	5.40–22.93	<0.001	1.02	0.69–1.52	0.919	1.06	0.71–1.58	0.761	0.09⁎	0.05-0.20	<0.001
Knowledge												
High	0.78	0.18–3.39	0.738	0.74	0.29–1.88	0.526	1.47	0.57–3.78	0.423	1.26	0.08–20.93	0.873
Medium	1.01	0.32–3.22	0.990	0.64	0.32–1.29	0.212	1.69	0.83–3.45	0.151	4.30	0.55–33.59	0.164
Low	Reference			Reference			Reference			Reference		

⁎ Firth logit analyses were done instead of regular logistic regression.

Farmer knowledge (categorized as high, medium, low) did not show significant associations with AMR or MDR outcomes. Both high and medium knowledge groups had odds ratios fluctuating above and below 1, but none were statistically significant. Similarly, to the univariable analyses, the multivariable analyses did not reveal any significant predictors to resistance in farm level samples (Table 5).

**Table 5:** Multivariable analysis of antimicrobial resistance (AMR) and multidrug resistance (MDR) predictors.

Predictors	AMR						MDR					
	AMR samples⁎ (either Gram± bacteria)			AMR samples (both Gram± bacteria)			MDR samples (either Gram± bacteria)			MDR samples⁎ (both Gram± bacteria)		
State	Odds ratio	95% CI	*p*-value	Odds ratio	95% CI	*p*-value	Odds ratio	95% CI	*p*-value	Odds ratio	95% CI	*p*-value
Haryana	0.54	0.09–3.34	0.505	0.49	0.18–1.31	0.155	0.92	0.35–2.43	0.87	0.41	0.08–2.05	0.276
Assam	Reference			Reference			Reference			Reference		
Milking cow	0.96	0.77–1.21	0.754	1.09	0.86–1.37	0.487	1.00	0.81–1.22	0.970	0.68	0.33–1.38	0.281
Cleanliness of cow	2.83	0.14–58.88	0.502	2.78	0.73–10.64	0.136	1.68	0.48–5.84	0.417	0.49	0.06–4.22	0.514
History of Mastitis	0.93	0.13–6.86	0.945	1.89	0.52–6.85	0.335	1.60	0.44–5.85	0.479	3.71	0.69–19.89	0.126
Antibiotic use	1.78	0.03–108.72	0.783	0.85	0.07–10.61	0.897	4.53	0.24–85.83	0.314	6.20	0.21–186.93	0.294
Knowledge												
High	0.69	0.09–5.50	0.726	0.87	0.18–4.24	0.864	0.98	0.21–4.53	0.977	0.33	0.05–19.37	0.594
Medium	2.20	0.30–16.06	0.438	1.67	0.42–6.62	0.466	1.75	0.46–6.63	0.410	2.21	0.11–43.21	0.600
Low	Reference			Reference			Reference			Reference		
Constant	8.52	0.82–88.23	0.072	0.81	0.175–3.73	0.785	0.68	0.15–3.00	0.611	0.19	0.006–5.86	0.342

⁎ Firth logit analyses were done instead of regular logistic regression.

## 4 Discussion

This study highlights the widespread occurrence of AMR in Indian milk samples, reflecting the complex and multifactorial nature of resistance in livestock systems. The prevalence of AMR was uniformly high, while MDR varied more by milk type and handling practices than by farm-level characteristics. These findings are consistent with earlier work showing that AMR in livestock is shaped by systemic and contextual factors rather than easily measurable single risk factors [41].

Our results showed that AMR prevalence was high in both Assam (91%) and Haryana (89%), with no significant difference between the states. This aligns with global reports documenting high AMR prevalence across multiple livestock settings [42], [43], [44]. Dual AMR was observed in nearly half of all samples (48%), similar to MDR prevalence (48%), while dual MDR remained relatively rare (7%). Farmer milk showed higher resistance levels compared to raw vendor-sourced milk, although not statistically significant, may suggest potential contamination risks in farm environment.

Although pasteurized milk samples were collected only from one state (Haryana) and the sample size was limited, resulting in wide confidence intervals, an important finding of this study was that pasteurized milk showed a significantly higher prevalence of dual multidrug resistance (23%) compared with raw milk (6%). This counterintuitive observation may reflect post-pasteurization contamination, persistence of resistant strains within processing environments, or inadequate handling and hygiene practices during packaging and distribution. Similar patterns of AMR bacteria in pasteurized dairy products have been reported previously [45]. Pasteurization significantly reduces microbial load, including pathogens such as *Salmonella*, *Listeria monocytogenes*, *S. aureus*, and *E. coli* O157:H7 [46]. However, studies have shown that residual resistance genes and post-pasteurization contamination can still allow resistant organisms such as *Pseudomonas* spp. and other Enterobacteriaceae spp. to persist [47], [48], [49]. Thus, while pasteurized milk carries fewer viable AMR bacteria compared to raw milk, its potential as a vehicle for antimicrobial resistance genes (ARG) dissemination remains a concern [50], specifically for consumers who prefers to drink milk without boiling.

No links could be found between knowledge level and AMR presence in the milk in this study. Socio-economic and behavioural aspects play a critical role in shaping antibiotic usage patterns in the dairy industry. Limited access to veterinary services often drives farmers to rely on non-prescription or over-the-counter antibiotics, leading to inappropriate use, sub-therapeutic dosing, and incomplete treatment courses [51], [52], [53]. Farmer knowledge, perceptions of antibiotic necessity, and economic pressures to maintain herd productivity further influence treatment decisions [54]. Consequently, interventions aimed at strengthening farmer education, antibiotic stewardship, and veterinary guidance are crucial for mitigating resistance at the farm level.

Our hypothesis was that the risk model created in the causal framework could help identify key predictors contributing to AMR in the study. While increased herd size and poor cleanliness practices were initially hypothesized as potentially important contributors, their associations with AMR could not be observed. A history of mastitis appeared as a potential, though statistically non-significant, risk factor. Importantly, farmer knowledge and awareness emerged as pivotal elements in potentially mitigating AMR risk, but this could not be verified in the statistical analyses. Even though we could not identify risk factors in this study, the guidance of the causal framework was helpful. The causal diagram can help design targeted strategies to reduce AMR by focusing on promoting better hygiene practices to reduce mastitis, enhancing knowledge among livestock farmers, optimizing antibiotic use to limit misuse or overuse.

In fact, our regression analyses revealed that most hypothesized predictors including state, herd size, mastitis history, antibiotic use, and farmer knowledge were not significantly associated with AMR or MDR. Similar null findings have been reported in studies examining herd-level factors such as mastitis and antibiotic use, where associations with resistance did not reach significance, often due to small sample sizes or unmeasured confounding [55], [56], [57]. Cleanliness of cows appeared to increase the odds of dual AMR in univariable models, but this effect lost significance in multivariable analysis and was characterised by wide confidence intervals. The unexpected association between reported cleanliness and higher AMR could indicate reverse causation, whereby farms with known AMR issues report stricter cleaning, but as the association disappeared in the multivariable model it was likely spurious. However, existing literature has earlier linked poor hygiene, environmental contamination, and inadequate sanitation with higher AMR prevalence [58], [59], [60], [61].

Given the rarity of dual MDR events, Firth's penalized logistic regression was applied. This method is recommended for small or imbalanced datasets as it reduces estimation bias and improves model stability [62]. Although Firth models produced effect estimates consistent with standard logistic regression, confidence intervals remained wide, indicating high uncertainty of the estimates.

Overall, the study confirms that AMR is deeply entrenched in dairy ecosystems in India, with little evidence that common farm-level predictors explain the observed resistance. Instead, contamination seems to be able to occur along the value chain. This study has few limitations. The relatively small number of pasteurized milk samples (*n* = 17) limited statistical power and generalizability. Self-reported variables may have been affected by recall or social desirability bias. The cross-sectional study design restricts causal inference and may not account for seasonal variation in AMR dynamics. While a higher prevalence of dual MDR was observed in pasteurized milk (23% vs. 6% in raw milk, *p* = 0.023), this finding is based on a limited sample size, and potential explanations such as post-pasteurization contamination, persistence of resistant strains in processing environments, or handling practices could not be confirmed. Additionally, pasteurized milk is typically sourced from organised cooperative sectors where antibiotic usage may differ from unorganized vendors, but our study did not allow for detailed evaluation of this. Finally, microbial source tracking and parallel human sampling were not conducted, preventing confirmation of contamination sources. Therefore, these findings should be interpreted with caution, and larger, more comprehensive studies are recommended to validate the observations.

## 5 Conclusion

This study demonstrates the presence of antimicrobial-resistant and multidrug-resistant bacteria in milk samples collected from both informal and more organised dairy production settings in India. Dual resistance in Gram-positive and Gram-negative bacteria was also observed. However, multivariable analyses showed largely null associations between the investigated farm- and vendor-level factors and AMR outcomes, and prevalence estimates were characterised by wide confidence intervals due to sparse data and imbalanced sample sizes. Accordingly, the findings should be interpreted as descriptive and hypothesis-generating. Larger, systematically designed studies with improved organism-level resolution and balanced sampling across the dairy value chain are required to more robustly identify drivers of antimicrobial resistance in Indian milk production systems.

## CRediT authorship contribution statement

**Tushar K. Dey:** Writing – review & editing, Writing – original draft, Visualization, Validation, Software, Methodology, Investigation, Formal analysis, Data curation, Conceptualization. **Garima Sharma:** Writing – review & editing. **Åke Lundkvist:** Writing – review & editing, Visualization, Supervision, Resources. **Delia Grace:** Writing – review & editing, Visualization, Supervision, Resources, Project administration. **Bibek R. Shome:** Writing – review & editing, Project administration, Funding acquisition. **Ram P. Deka:** Writing – review & editing, Project administration. **Rajeswari Shome:** Writing – review & editing. **Samiran Bandyopadhyay:** Writing – review & editing. **Naresh K. Goyal:** Writing – review & editing. **Johanna F. Lindahl:** Writing – review & editing, Visualization, Validation, Supervision, Resources, Project administration, Methodology, Investigation, Funding acquisition, Data curation, Conceptualization.

## Declaration of competing interest

The authors declare that they have no known competing financial interests or personal relationships that could have appeared to influence the work reported in this paper.

## Data Availability

Data is made available from the authors upon reasonable request.

## Appendix A Supplementary data

Supplementary material 1 Image 1

Supplementary material 2 Image 2
