# Supplementary material for: Antimicrobial resistance in India's dairy value chain: An investigation of risk factors in Haryana and Assam, India
Source: One Health. 2026 Mar 20;22:101391. doi: 10.1016/j.onehlt.2026.101391 (PMC13050009; doi:10.1016/j.onehlt.2026.101391)
Supplement: Supplementary file 1 — Supplementary material 1 [file mmc1.docx]

**Questionnaire**

**For assessing farming system, risk factors and economic losses caused brucellosis and antibiotic resistance**

1. **Name and address of the respondent NB: must be responsible for bovine health decisions**

|  | Name of interviewer | interviwr |  | Date | date |
| --- | --- | --- | --- | --- | --- |
|  | North | Longitude |  | East | latitude |
|  | Name of the HH head | hh_name |  | Phone no. | phone |
|  | Village/Ward | villag |  | Block/Town | block |

1. **Basic demography & farm details**

|  | Gender of respondent (1=male, 0=female) | gendr |  | No of HH members | Hh_memb |
| --- | --- | --- | --- | --- | --- |
|  | Highest education (0= No education, 1=primary, 2=class 5-10, 3=Higher secondary, 4=Graduation and above) | educatn |  | Age (years) | age |

|  | Have any member of your family ever availed training on livestock management (Yes=1, No=0) | traini_live |
| --- | --- | --- |
| 2.6 | Did you ever receive training on animal disease? (1=Yes, 0=no) | Traini_disea |
| 2.6.1 | If yes, who gave it? | who_gave |
| 2.6.2 | If yes, did you learn about giving medicines to animals? | Learn_medi |
| 2.6.3 | Do you hand milk or use machine? (Hand milking =1, machine milking =2) | Hand_mach_milk |
| 2.6.4 | How many persons do milking at your farm? | Pers_milk |
| 2.6.5 | Do all involved in milking live in the same household? (1=Yes, 0=no) | Live_samehh |

1. **Herd size**

| **Sl. No.** | Species | milking | dry cows/ buffalo cows | adult bulls/bullock | heifer | Calves |
| --- | --- | --- | --- | --- | --- | --- |
|  | Cattle | **Catl_milk** | Catl_dry | Catl_bull | Catl-heif |  |
|  | Buffalo | **Buff_milk** | Buff_dry | buff_bull | buff_heif |  |

1. **Other species present on the farm**

| Other species in farm | | | | Goat | Sheep | Pigs | Dogs | Cats | Poultry | | Other:___________ |
| --- | --- | --- | --- | --- | --- | --- | --- | --- | --- | --- | --- |
| Number goat_no | | | | **ggno** |  |  |  |  |  | | Other_no, other_cont |
| Can this species (even if not your animal) get in contact with the dairy animals (Yes=1, No=0) goat_cont | | | |  |  |  |  |  |  | | Other_cont |
|  | | Do your dairy animals come into contact with wild / jungle animals like rats, wild boars, wild buffalo, monkeys, bats etc.(1= yes, 0=no) | | | | | | | | conta_wildanim | |
|  | | Do you move your animals from one place to another in search of feed/grazing/market/breeding (take cows to bull/AI, or take bull to other farm)? (1=yes, 0=no) | | | | | | | | animl_mov | |
|  | | Rearing system (1=fully stall fed/ tied stall, 2= partly stall fed(grazing part time), 3=no stall feeding (only grazing)) | | | | | | | | reari_systm | |
| **Sl. No.** | | **Parameters** | | | | | | |  | |  |
|  | | Breeding is done by ( 1= Own bull kept at the farm, 2= natural mating by a bull brought to the farm or community bull, 3= mating while common grazing 4= Artificial Insemination ) Multiple options allowed | | | | | | | Breedi_ownbul, breei_brought  Mating_grazing | |  |
|  | | How many new animals introduced in **last 12 months**? | | | | | | | Animl_intro | |  |
|  | | Source of new animals (1= known herd, 2= unknown herd 3=Government source) | | | | | | | Sourc_intro | |  |
|  | | Do you follow specific practices/ quarantine for newly brought animal before introducing to your herd (1=yes, 0=no.) | | | | | | | quarantine | |  |
|  | | If yes, describe what you do and for how long: | | | | | | | Describ_quart | |  |
|  | | Have you **ever** bought an animal you knew was weak but cheap ( 1= yes, 0=no) | | | | | | | Weak_cheap | |  |
|  | | Have you **ever** had an animal get sick shortly after purchase (1=yes, 0=no) | | | | | | | Sick_purch | |  |
|  | | Do you clean/wipe the udder before milking? (0=never, 1=Sometimes, 2= Always) | | | | | | | clean_udr | |  |
|  | | If you do, describe what you do: | | | | | | | Describ_clean | |  |
|  | | Do you use disinfectant after milking? (0=never, 1=Sometimes, 2= Always) | | | | | | | disinf_milking | |  |
|  | | How much water is one milking cow/buffalo cow provided per day on average (in lit/day)? **(If don’t provide –write 0)** | | | | | | | water_cow | |  |
|  | | How much concentrate/feed ingredients do you give per milking cow per day? **(If don’t buy- write 0)** | | | | | | | concent_cow | |  |
|  | | What is the price of per kg concentrate/feed ingredient? In rupees | | | | | | | Concent_pric | |  |
|  | | How much green grass do you give per milking cow per day?**(If don’t give- write 0)** | | | | | | | Grass_cow | |  |
|  | | What is the price of per kg green grass? In rupees | | | | | | | grass_pric | |  |
|  | | How much dry feed do you give per milking cow per day?**(If don’t give- write 0)** | | | | | | | dryfee_cow | |  |
|  | | What is the price of per kg dry feed? In rupees | | | | | | | dryfee_pric | |  |
|  | | How much do you pay for insurance cover per animal? **(If don’t have- write 0)** | | | | | | | Insurpric_cow | |  |

1. **Milk production and reproduction details**

| **Sl. No.** | **Parameters** | **Response** |
| --- | --- | --- |
|  | How much milk did your farm produce yesterday (litres)? | Milk_prod |
|  | How much milk was consumed in the household yesterday (litres)? | milk_consu |
|  | How is milk consumed? 0=Don’t drink milk 1=Boiled; 2=processed (curd, butter, cottage, sweets etc) 3= raw, 4= pasteurized , **multiple answers allowed** | Ho_consu_boil  Milk_procesed  Milk_raw |
|  | How much milk (in litres) did you sell yesterday? | Milk_sold |
|  | Selling price/lit (Rs.) | Milk_pric |
|  | **Think about the last time you had a heifer that had a calf:** |  |
|  | How old (in months) was she when you mated her the first time? | Mated_first |
|  | How old (in months) was she when she had her first calf? | Age_firstcal |
|  | After she had a calf, how many months did you wait before you mate her the next time? | Mated_second |
|  | What is the price (Rs.) of an artificial insemination? | Pric_ai |
|  | What is the price (Rs.) of a mating with a bull? | Pric_natmat |
|  | What is the value of a male calf (6 months age)? | Pric_malcalf |
|  | What is the value of a female calf (6 months age)? | Pric_femcalf |

1. Picture card

|  |  | a | b | c | d | e | f | g | h | i |
| --- | --- | --- | --- | --- | --- | --- | --- | --- | --- | --- |
| 6.1 | Do you recognize this medicine? (1=Yes, 0=no) |  |  |  |  |  |  |  |  | Recon_med |
| 6.2 | What is its name? |  |  |  |  |  |  |  |  | Name_med |
| 6.3 | Did your cows have this medicine in the last 12 months? |  |  |  |  |  |  |  |  | usemed_year |
| 6.4 | For which reason was it used? |  |  |  |  |  |  |  |  | Use_reasn |

1. Knowledge and practice on antibiotic use

| 7.1 | Have you heard about antibiotics? (1=Yes, 0=no) | Heard_antbiot |
| --- | --- | --- |
| 7.2 | **If yes,** Please describe what antibiotics do:  descrb_antibot | |
| 7.3 | Have you heard of withdrawal period (1=Yes, 0=no) | Heard_withdrl |
| 7.4 | **If yes,** Please describe it : |  |
| 7.5 | When do you stop treating an animal with a medicine?  (1= when I see it is recovering OR looks healthy  3= Continue as long as veterinary says 4= When I don’t have more money/medicine to treat) | Stop_treatit |
| 7.6 | Where are medicines discarded?  (0= throw away outside 1= garbage bin 2= Return to seller 3= burn 4= Bury  5= drain 6= don’t bother 7= others:___________) | Med-discrd |

1. Knowledge and attitude towards antibiotic use

|  | Do you agree with the following:  If farmer has not heard about antibiotics use medicine instead of antibiotic | agree=1,/  disagree=0/  Don’t know=99 |
| --- | --- | --- |
| 8.1 | The more an antibiotic/medicine costs, the better it is | Moreantb_bett |
| 8.2 | All antibiotic/medicine suitable for people can be used in animals | Antb_menanim |
| 8.3 | If the milk from a treated cow looks normal it is ok to consume | Milknorm_cons |
| 8.4 | The pharmacist is as good as the vet to decide which antibiotic/medicine | Pharm_good |
| 8.5 | All diseases need treatment | Dises_treat |
| 8.6 | Injections are always more powerful than oral antibiotic/medicine | Injet_pwerf |
| 8.7 | There are many poor quality antibiotic/medicine on the market | Antb_porqult |
| 8.8 | Increasing the amount of antibiotic/medicine will make it more effective | Increa_antbi |
| 8.9 | Antibiotics stop all diseases | Antibi_aldises |
| 8.10 | If one cow is sick, then others should be treated also to prevent disease | Onecow_tretoth |
| 8.11 | Antibiotics can cure foot and mouth disease | Antib_fmd |
| 8.12 | If using antibiotic too often, it may stop being effective | Antib_stopwork |

1. **Now we wonder about if you take some preventive measures in your herd.**

|  | Have you vaccinated your animals in the last 12 months? (1=yes 0=no) | | vaccinate |
| --- | --- | --- | --- |
|  | If yes, do you know against which diseases the animals have been vaccinated? (1=yes 0=no) | | Yes_no |
|  | Name those, if yes: | | Name_yes |
| - 1. Do you use disinfectant (Dettol, bleach, potash etc.) in cleaning the farm? (1= yes 0=no) | | | Disinftn_yn |
| - 1. Frequency of disinfection: 1= daily, 2= at least 1/week, 3= at least every month, 4=more seldom | | | Freq_disinftn |
| - 1. When you treat your animals with medicines, what do you do with the milk during those days?   1. Sell 2. Throw away 3. Keep in the household for consumption  4. Offer to calves 5. Others, specify: ___________________________ | | | usemilk_treat |
| - 1. What do you do with the milk of a sick animal which is not treated?   1. Sell 2. Throw away 3. Keep in the household for consumption 4. Offer to calves 5. Others, specify: ____________________________ | | | Usemilk_sick |
| - 1. What do you do with the placenta after a normal calving? | | 1. Throw away 2. Bury  3. Burn 4. Offer to dogs to eat 5. Other, specify_____________ | Plactnt_normcalvi |
| - 1. How often do you have veterinary consultations? | | 0=Never, 1= weekly (more than once per week), 2=monthly (more than once per month), 3= yearly, 4= more seldom | Vetconslt_freq |

**Knowledge and attitude about brucellosis**

| 1. **Do you know what brucellosis is?** | 1. I know what it is  2. I have heard the term brucellosis but do not know what it is  3. I don’t know anything about brucellosis **(in that case go to question 17**) | Know_bruce |
| --- | --- | --- |
| 1. **Do you know who can be affected with brucellosis?** | 1. Cattle 2. Buffalo 3. Goats 4. Sheep 5. Dog/rodent 6. Humans 7. Pig 8. Don’t know **Multiple options allowed** | Who_afect |

- 1. If yes for humans, what symptoms of brucellosis in humans, do you know?
  2. Which symptoms are you expecting from your animals when they get it?

| 1. **Do you know if there is any Brucella vaccine? Multiple options allowed**   1. Yes, for animals 2. Yes, for humans 0. No | **Know_brucvac** |
| --- | --- |
| 1. **Brucellosis is transmitted from (Multiple options allowed)** 1. Animal to human 2. Human to animal, 3. Human to human 4.Animal to animal 5. Wild animals to domestic animals 6.Don’t know | Bruc_tranm |
| 1. **Can the consumption of raw milk/milk products transmit brucellosis?**   1. Yes 0. No 2. Don’t know | Rawmilk_transm |
| 1. **Do you think brucellosis can be transmitted in a farming household, like yours?** 1. Yes 0. No 2. Don’t know | **Transm_farmhh** |
| 1. **Has brucellosis been diagnosed (in laboratory) among your animals in the last 12 months?** 1. Yes 0. No 2. Don’t know | **Diagn_anm** |

1. Cattle illness in the preceding 12 months

| Sl.No | Disease/ symptom/ condition | | How many animals got this last 12 months? **99= don’t know, 0= No animals** | Trend in last 12 months from previous years  **1= More this year; 2= less this year; 3= no change** | How many animals got treated with medicine last 12 months?  **99= don’t know, 0= No animals** | Name of medicine or type of medicine given for this last time a cow got sick. |
| --- | --- | --- | --- | --- | --- | --- |
| 17.1 | Mastitis | |  |  |  |  |
| 17.2 | Respiratory | |  |  |  |  |
| 17.3 | Diarrhea | |  |  |  |  |
| 17.4 | Lameness | |  |  |  |  |
| 17.5 | Fever | |  |  |  |  |
| 17.6 | abnormal vaginal discharge (pus, strange color or odour) | |  |  |  |  |
| 17.7 | Repeat breeding | |  |  |  |  |
| 17.8 | Abortion | |  |  |  |  |
| 17.9 | Retention of placenta | |  |  |  |  |
| 17.10 | Still birth | |  |  |  |  |
| 17.11 | Male infertility | |  |  |  |  |
| 17.12 | Carpal hygroma | |  |  |  |  |
| 17.13 | Any other specific disease you want to mention: | |  |  |  |  |
|  | | 17.14 Did any cow have an allergic reaction after treatment? (1=Yes, 0=no)  17.15 **If yes,** How many last 12 months? | | | | |

1. **About the abortion**

**If no abortions last year, go to question 25**

| **S.l No.** | | | **Parameters** | | **Response** |
| --- | --- | --- | --- | --- | --- |
|  | | In which stage of pregnancy, abortion occurred last time  (1= first trimester, 2= 2^nd^ trimester, 3= 3^rd^ trimester) | | |  |
|  | | How does the aborted foetus/ placenta looked last time  (1= fresh and moist, 2=leathery and dry) | | |  |
| - 1. **What do you do with the placenta and foetus, if aborted?** | | | | 1. Throw away 2. Bury  3. Burn 4. Offer to dogs to eat 5. Other, specify____________________ |  |
| - 1. **Do you take any protection while you handle aborted foetus/ aborted materials? Multiple options allowed.** | | | | 1. Wear gloves 2. Wash hands with soap  3. wash hands without soap  4. Get the clothes washed  5. Take bath 6. Nothing/ don’t care  7: Other, specify……….. |  |
|  | Did any cow/buffalo die after abortion? **If no, go to question 21** | | | |  |
|  | What would have been the price of this animal if you had sold in the market instead of dying? | | | |  |
|  | How many less litres per day did you get because of the abortion? | | | |  |
|  | For how many days was milk reduced? | | | |  |
|  | How much money did you spend on medicine after the last abortion? | | | |  |
|  | How much did you spend on paying a doctor/veterinarian after the last abortion? (Rs) **(If nothing, write 0)** | | | |  |
|  | How much money did you spend on indigenous treatment for reproductive problems (RS) the last 12 months? **(If nothing, write 0)** | | | |  |

1. **Have you experienced any other loss caused by reproductive diseases in last 12 months?**

1. Yes 0. No

**If not, go to the observation check list.**

| **S.l No.** | **Items of losses** | **Response** |
| --- | --- | --- |
|  | How many animal had difficulties to get pregnant in last 12 months? |  |
|  | If yes, how many inseminations/matings were needed for pregnancy in these animals in total for all? |  |
|  | Did you cull/sell any animal after suffering from reproductive disease (abortion/repeat breeding/ retention of placenta etc.)? (1=yes, 0=no) |  |
|  | Which price did you sell this cow for? |  |
|  | Which price could you have gotten if the cow was healthy? |  |
|  | If you kept a cow with reproductive problems, how many days of full production do you estimate that you lost due to this? |  |

**Observation check list**

G Farming system details

|  | **Parameters** | **Response** |
| --- | --- | --- |
| G.1 | Floor type (0=Bricks, 1=concrete, 2= Earthen, 3= other (specify)) |  |
| G.2 | Cleanliness of house (1 = very, 2 =some, 3 = dirty) |  |
| G.3 | Roof: 1= Pakka/concrete slab 2= Thatched roof 3= corrugated tin/asbestos, 4= traditional roofing tiles, 5= concrete tiles, 6= bamboo corrugated sheet 7= other………………… |  |
| G.4 | Separate houses for: 1= calves, 2= Pregnant, 3= Sick (Multiple options allowed) |  |
| G.5 | Cleanliness of cows (1=very, 2=some, 3=not) |  |
| G.6 | Medicine storage (0= no storage 1=Open and used packages stored, 2=sealed cabinet, 3=exposed to sun, 4= nothing specific, 5= store house/cattle shed) Multiple options allowed |  |
| G.7 | Are there discarded medicines, wrappers, syringes on farm premises (1=yes, 0=no) |  |

H. Medicine list: **Make a list of all the medicines on the farm including any medicated food and discarded packages Containing antibiotics. If unsure, list it.**

| Medicine name | Quantity | Expiry date | open/closed package | What farmer uses for | How much used in last 12 months | Where or from whom did you buy this pack? |
| --- | --- | --- | --- | --- | --- | --- |
|  |  |  |  |  |  |  |
|  |  |  |  |  |  |  |
|  |  |  |  |  |  |  |
|  |  |  |  |  |  |  |
|  |  |  |  |  |  |  |
|  |  |  |  |  |  |  |
|  |  |  |  |  |  |  |
|  |  |  |  |  |  |  |
